# Operational characteristics of 30 lateral flow immunoassays used to identify COVID-19 immune response

**DOI:** 10.1016/j.jim.2021.113096

**Published:** 2021-09

**Authors:** Anura David, Lesley Scott, Sarika Jugwanth, Maemu Gededzha, Trish Kahamba, Nontobeko Zwane, Nakampe Mampeule, Ian Sanne, Wendy Stevens, Elizabeth S. Mayne

**Affiliations:** a Department of Molecular Medicine and Haematology, School of Pathology, Faculty of Health Sciences, University of Witwatersrand, Johannesburg, South Africa; b National Health Laboratory Services, Johannesburg, South Africa; c Department of Immunology, Faculty of Health Sciences, University of Witwatersrand, South Africa; d Health Economics and Epidemiology Research Office, Department of Internal Medicine, School of Clinical, Medicine, Faculty of Health Sciences, University of the Witwatersrand, Johannesburg, South Africa; e Clinical HIV Research Unit, Department of Internal Medicine, School of Clinical Medicine, Faculty of Health Sciences, University of the Witwatersrand, Johannesburg, South Africa

**Keywords:** SARS-CoV2, COVID-19, Lateral flow immunoassays, Point-of-care

## Abstract

Serology or antibody tests for COVID-19 are designed to detect antibodies (mainly Immunoglobulin M (IgM) and Immunoglobulin G (IgG) produced in response to Severe Acute Respiratory Syndrome Coronavirus 2 (SARS CoV-2) infection.

In this study, 30 lateral flow immunoassays were tested using serum or plasma from patients with confirmed SARS CoV-2 infection. Negative serological controls were accessed from a well-characterised bank of sera which were stored prior to February 2020. Operational characteristics and ease of use of the assays are reported.

4/30 (13%) of kits (Zheihang Orient Gene COVID-19 IgG/IgM, Genrui Novel Coronavirus (2019-nCoV) IgG/IgM, Biosynex COVID-19 BSS IgG/IgM, Boson Biotech 2019-nCoV IgG/IgM) were recommended for SAHPRA approval based on kit sensitivity. Of these, only the Orientgene was recommended by SAHPRA in August 2020 for use within the approved national testing algorithm while the remaining three received limited authorization for evaluation. All kits evaluated work on the same basic principle of immunochromatography with minor differences noted in the shape and colour of cartridges, the amount of specimen volume required and the test duration.

Performance of the lateral flow tests were similar to sensitivities and specificities reported in other studies. The cassettes of the majority of kits evaluated (90%) detected both IgG and IgM. Only 23% of kits evaluated contained all consumables required for point-of-care testing. The study highlights the need for thorough investigation of kits prior to implementation.

## 1 Introduction

The Severe Acute Respiratory Syndrome Coronavirus 2 (SARS CoV-2) originated in Wuhan, Hubei Province, China towards the end of 2019 and was declared a global pandemic in March 2020 by the World Health Organization (Cucinotta and Vanelli, 2020). The initial cases in South Africa were described in early March 2020 and a nationwide lockdown was instituted on the 23 March 2020. This notwithstanding, there was rapid rise in infection rates in South Africa with over 775,000 infections confirmed by mid-November 2020.

Coronaviruses are enveloped viruses with a lipid membrane derived from the host cell, in which viral surface proteins are embedded. These include nonstructural proteins such as the RNA-dependent RNA polymerase (RdRp), and essential structural proteins, namely the nucleocapsid (N) protein and membrane proteins: the S (spike) -glycoprotein, the matrix (M) protein, and the envelope (E) protein (Fehr and Perlman, 2015; Ludwig and Zarbock, 2020). One or more of these proteins usually represent the target regions of most diagnostic molecular assays.

Diagnostic testing for SARS-CoV2 is critical to identification of infected individuals allowing isolation and interrupting transmission chains. The testing pipeline comprises 2 major test types – molecular and antigen testing and serological (antibody testing). For molecular testing, the reverse-transcriptase polymerase chain reaction (RT-PCR) test is the mainstay of diagnosis particularly at early time-points post-symptom onset but the sensitivity may be reduced at later time-points in the disease course reference.

Serology or antibody tests for coronavirus disease of 2019 (COVID-19) are designed to detect antibodies (mainly Immunoglobulin M (IgM) and Immunoglobulin G (IgG)) produced in response to SARS CoV-2 infection. These tests have been designed for both high-throughput platforms and for point-of-care applications. Previous experience with serological assays in testing for other Coronavirus species has highlighted that these tests are important in epidemiological surveys, tracing sources of infection and for patient contact studies (Meyer et al., 2014). Although the humoral response to SARS-CoV-2 currently is incompletely defined, it appears that approximately 60% of infected individuals produce IgM antibodies from about day 4 post-symptom onset. IgM levels peak between day 14 and 21 and then decline. IgG levels begin to rise at about 7–14 days peaking around day 25. It is unclear how long IgG levels are sustained although some individuals had detectable IgG antibodies 2–3 months after onset (Liu et al., 2020). There are currently >350 tests that are either commercially available or under development (FIND, 2020). Test types include formal laboratory based assays including ELISA assays (Van Elslande et al., 2020) and point-of-care (POC) rapid lateral flow immunoassays (LFIA) (Wu et al., 2020), chemiluminescent immunoassays (CLIA) (Bastos et al., 2020) and neutralization assays (Ghaffari et al., 2020).

Lateral flow immunoassays are paper-based platforms where a liquid specimen (typically whole blood, serum or plasma in the case of serology) containing the analyte of interest moves through capillary action through various zones of polymeric strips, on which molecules that can interact with the analyte are attached (Koczula and Gallota, 2016). If the analyte of interest is present in the specimen, the presence of a test line is seen within 5–30 min. LFIAs require minimal laboratory space and equipment, can be easily interpreted, and can be performed with minimal training by testing personnel. This makes these assays attractive for field testing particularly in remote sites with limited laboratory access. A number of these tests have been marketed for SARS CoV-2 although there is substantial variability between different tests including the contents of the kits, specimen volume required, and other operational characteristics such as the ease of use, the test duration and the specimen type required. Rapid SARS-CoV2 tests have been developed across the world but have also shown markedly variable performance resulting in recalls in some regulatory jurisdictions including the Food and Drug Administration of the United States (FDA, 2020).

In South Africa, the National Health Laboratory Service (NHLS) is the primary supplier of pathology services to the state healthcare sector (80% of the population). It also serves a reference and advisory service to the South African Health Products Regulatory Authority (SAHPRA) in the test licensing process. It is, therefore, responsible for assessing test performance of assays for implementation in South Africa including those assays which can be utilized for near or at-patient care. The Immunology Laboratory at NHLS Braamfontein is an ISO15189:2012 accredited facility with expertise in infectious disease serology testing. In collaboration with Clinical Laboratory Services (Department of Molecular Medicine and Haematology, Johannesburg, South Africa) [CLS], the laboratory undertook to evaluate all LFIAs, that received SAHPRA-approval for import, in order to assist the regulatory authority with licensing decisions and also to investigate the clinical utility and ease of use of these assays.

## 2 Materials and methods

This study was approved by the Human Research Ethics Committee of the University of the Witwatersrand (M200468). Participants who tested positive by RT-PCR for at least 2 genes were requested to participate in the study and then enrolled, following informed consent (*n* = 587). Bloods were collected at a single time-point for each participant. An average of 150 samples were tested post-PCR and care was taken to ensure that representative samples of a pre-defined panel was used for each kit evaluated. Clinical staff collected study specimens while adhering to standard health and safety precautions required when handling confirmed and suspected cases of COVID-19 (FDA, 2020; Denning et al., 2020). Participants were requested to provide 2 × 6 mL Ethylenediamine tetraacetic acid (EDTA) tubes of blood, 4 × 4 mL serum separator tubes for serum storage, a nasal swab and saliva and to complete a questionnaire containing information on symptoms. Upon receipt at the testing laboratory (CLS) biorepository, specimens were immediately processed, sub-aliquoted and stored frozen at −80 °C. Freeze-thaw cycles were limited to one. The median time between sampling and onset of symptoms was 35 days (range: 10–60 days).

### 2.1 Positive controls

Patients infected with the SARS CoV-2 virus were recruited from i) clinical facilities admitting and isolating infected individuals, ii) volunteers at various clinical centres and iii) among those tested at various NHLS facilities in Gauteng and the Western Cape. Patients were stratified according to the number of days post-symptom or post-PCR diagnosis.

### 2.2 Negative controls

Specimens were collected from contacts of participants with confirmed negative real-time polymerase chain reaction (RT-PCR) results, and derived from a well-characterised bank of sera which were stored prior to February 2020. In addition, residual stored sera were utilized from individuals with confirmed autoimmune diseases with high polyspecific antibody production including rheumatoid arthritis and systemic lupus erythematosus and serum samples from patients with pneumonia of viral aetiology prior to 2020.

### 2.3 Reference assays

In this evaluation, two independent reference standards were utilized. PCR testing as conducted for initial diagnosis was utilized as an initial standard. PCR positivity was determined by RT-PCR laboratory testing and confirmed through an independent database. Where possible, the PCR test was repeated to confirm the initial result. A serological ELISA-based assay (EUROIMMUN Medizinische Labordiagnostika AG, Germany) was utilized as a secondary serological reference methodology. The EUROIMMUN assays were two of the first antibody detection tests to be validated and available for COVID-19 testing and uses the recombinant S1 domain of the SARS-CoV-2 spike protein as antigen. Formal serology was provided for reference purposes and did not form a component of the analysis for performance.

### 2.4 Evaluation protocol

LFIAs were recommended for evaluation after review of a technical dossier by SAHPRA. The majority of kits were donated for evaluation purposes.

All LFIAs were evaluated using a standardized protocol in 2 phases. An initial evaluation was undertaken utilizing 25 positive controls and 25 negative controls. For tests which met the acceptance criteria (defined as a sensitivity ≥85% and a specificity ≥98%) for IgG detection, a secondary evaluation testing an additional 100 specimens was undertaken.

### 2.5 Evaluation procedure

**Fig. 1:**
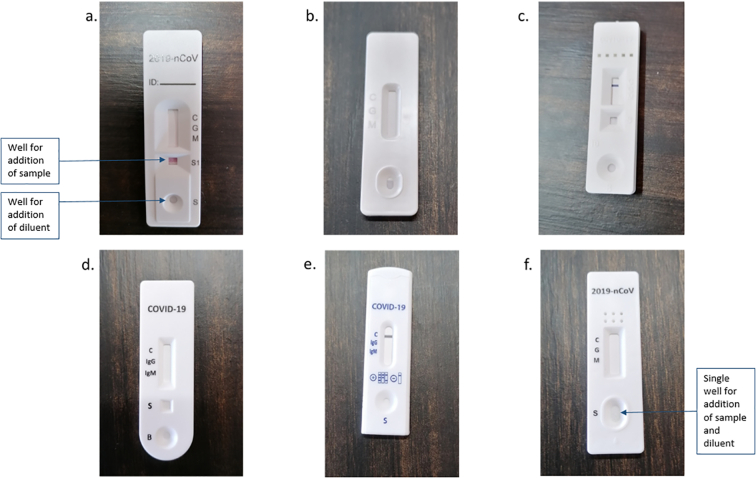
Examples of test cassettes.

The LFIA cassettes use whole blood (venous and/or fingerprick), serum or plasma to detect the presence of antibodies (IgG or IgM) produced against a specific SARS-CoV-2 antigen. Examples of the some of the LFIAs evaluated are shown in Fig. 1. All lateral flow cassettes evaluated contained a conjugate pad with recombinant COVID-19 antigens conjugated to colloidal gold (COVID-19 conjugates) and IgG and IgM-colloidal gold conjugates. Once diluted serum, plasma or whole blood is applied to the conjugate pad section, IgM and/or IgG antibodies, if present, will bind to COVID-19 conjugates to form an antigen-antibody complex. The complex then moves across the nitrocellulose membrane by capillary action. When the complex meets the line of the corresponding immobilized antibody (anti-human IgM and/or anti-human IgG), it is trapped to form a coloured band, which indicates a positive test result. The absence of a coloured band in the test region indicates a negative test result. The test contains an internal control band (C band) which should produce a coloured band for each test. This control line should be visible after the detection step as this confirms that the kit is working as intended. For the purpose of evaluation, plasma or serum was used and testing was performed as per package insert instructions. Minimal training was required for the testing procedure. Briefly, tests were performed by applying the serum or plasma specimen to the sample well of the cassette followed by the addition of specimen diluent. For the purposes of uniformity, a micropipette was used to ensure that the correct volume of specimen was added to each well. At the time of application, a timer was started and the strip was observed for the formation of control and test lines, following the incubation period recommended by the manufacturer.

C denotes control, G denotes IgG, M denotes IgM

a) Example of a cassette where the Specimen (S) and Sample diluent (S1) are added to separate wells. The cassette also has a defined space to record the patient ID.
b) and c) Examples of test cassettes where the markings on the cassette are the same colour as the cassette
d) Example of a cassette which has a different shape
e) Example of a test cassette which has a different shape and different coloured markings. This cassette also provided a brief interpretation of results
f) Example of the most commonly occurring cassette format

### 2.6 Acceptance criteria

Statistical analysis was performed using Stata version 16 (StataCorp LLC, College Station, TX, USA). Test performance for each of the 30 kits was measured by assessing the sensitivity, specificity and/or kappa co-efficient of the IgG LFIA result against PCR and formal serology. In addition to analytical performance, a desirable and acceptable target product profile was identified which comprised shipping and storage temperature, whether the kit detects IgG and/or IgM, specimen type required, recommended setting for use (laboratory or primary healthcare facility), test duration, kit contents and ease of use. To ensure that all kits were evaluated in a fair manner, all testing was performed using a standardized protocol, specimen type and panel. To determine performance, the testing panel used fulfilled World Health Organization (WHO) criteria to include majority (96%) of patients >21 days post onset of symptoms and also included specimens with different days of onset since illness. IgM was not used as an evaluation criteria since tests were conducted on specimens from individuals with a median of 35 days post-symptom onset when IgM levels were expected to be reduced, the formal comparative assays did not detect IgM and IgM is not produced by a proportion of infected individuals (Denning et al., 2020).

## 3 Results

### 3.1 Test performance

Only 4% of specimens tested were representative of the <14 day post-PCR category, hence data was not aggregated for performance analysis. 11% of participants tested were asymptomatic. For IgG, sensitivity of the kits in comparison to PCR and formal serology, ranged from 17% (95% CI: 4.74–37.4) to 96% (95% CI: 79.6–99.9) and 17% (95% CI: 4.74–37.4) to 100% (95% CI: 94.5–100), respectively with specificities ranging from 72% (95% CI: 50.6–87.9) to 100% (95% CI: 95.2–100) and 63% (95% CI: 44.9–78.5) to 100% (95% CI: 90.5–100), respectively (Table 1). Concordance between PCR and the LFIAs was also assessed using the kappa statistic which demonstrated values of 0.170 (95% CI: 0.014–0.326) to 0.954 (95% CI: 0.866–1.043). Three kits produced invalid results (no control line developed), the first demonstrated an invalid rate of 6/50 (12%); the second demonstrated an invalid rate of 8/96 (8%) and the third demonstrated an invalid rate of 1/50 (2%). For evaluation purposes, the specimen dropper from each kit (where available) was tested to determine ease of use. The majority of droppers (75%) proved difficult to use and the micropipette was preferred.

**Table 1:**
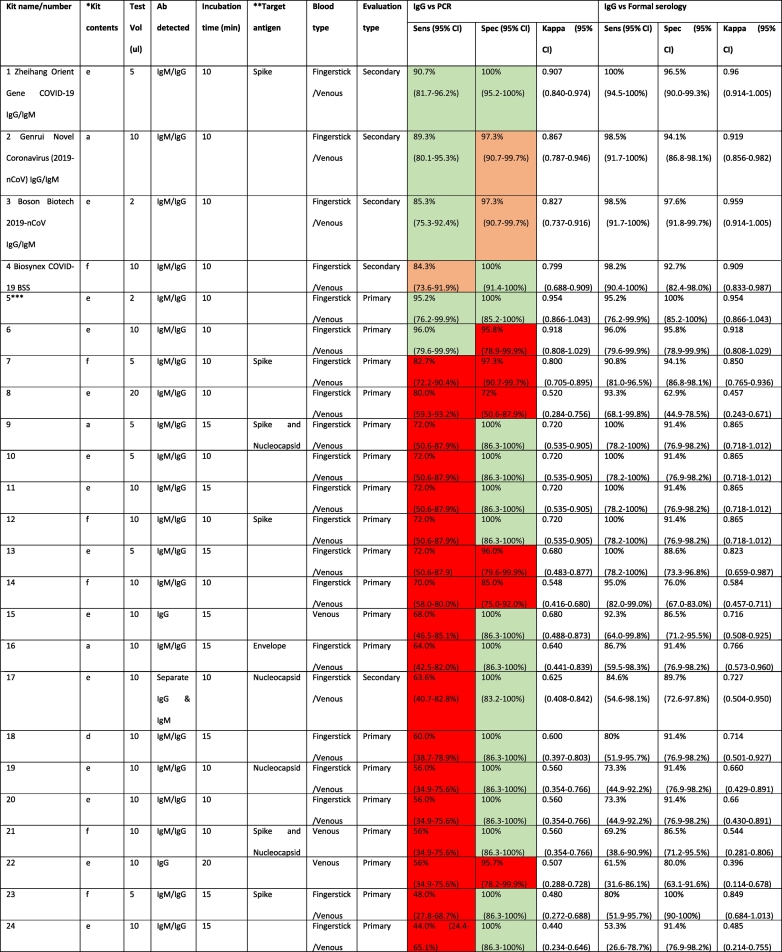

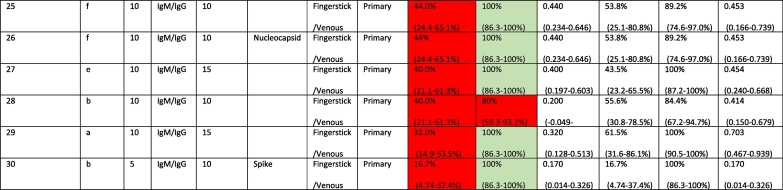
Summary of performance and operational characteristics of LFIAs evaluated.

*Kit contents key:

a - lancet, alcohol pad, dropper

b - dropper, lancet, alcohol pad

c - dropper and lancet

d - alcohol pad and lancet

e - dropper only

f - none

** Target antigen provided if available in published literature and/or package insert

*** Kit demonstrated high invalid rate (12%)

Red denotes data where acceptance criteria was not met, orange denotes data where SAHPRA allowed limited field validation and green denotes data where acceptance criteria was met

### 3.2 Time to positivity

19/30 (63%), 10/30 (33%) and 1/30 (3%) of kits required 10, 15 and 20 min of incubation, respectively. The median response time for a positive visual signal was also recorded. Some kits demonstrated a shorter time to positivity than others with a median time of 270 s (range: 15–943 s).

### 3.3 Operational considerations

Once the pouch containing the test cassette is opened, testing needs to be performed within 30–60 min. Of the 30 kits, 27 (90%) detected both IgG and IgM on one cassette (Table 1). One kit had separate cassettes for IgG and IgM and two kits detected IgG only. All kits contained a control line which served as a procedural control. 29/30 (97%) kits contained a package insert. Information on the target antigen was not available or could not be found for 63% (19/30) kits.

### 3.4 Kit shipping and storage

1/30 (3%) of kits required cold-chain transportation which will prove difficult in areas which are remote from distribution points, where these kits will most likely be used. All other kits could be transported and stored at 2–30 °C.

### 3.5 Kit contents

For the majority of kits, the end-user will need to provide their own lancets, alcohol pads and specimen droppers (Table 1). 3/30 (10%) could be tested on venous blood only, the alcohol pads and lancets were therefore not included in the kits. 3% (1/30) had individual specimen diluent ampules. The cassettes were similar in design, with minor differences in the shape, colour and size of cassettes. Other differences noted is that the labelling of test regions (IgM, IgG and C) on some cassettes were a different colour to the cassette and hence easier to read than others where the colours were the same. One kit contained cassettes in a bright blue colour which clearly distinguished this cassette from the others.

## 4 Discussion

Lateral flow or point-of-care serology tests represent a useful analytical tool for SARS-CoV2 detection. This is particularly true in African settings where clinics may be remote from laboratories. There has been, however, inconsistent performance resulting in kit recalls in many centers (FDA, 2020). This necessitates thorough evaluation prior to selection implementation of any specific product. Although multiple reviews of LFIA performance have been published to date (Bastos et al., 2020; Rashid et al., 2020; Kubina and Dziedzic, 2020), current review of the literature demonstrates that studies where the assays were physically evaluated range between two and nine LFIAs per study (Van Elslande et al., 2020; Wu et al., 2020; GeurtsvanKessel et al., 2020; Nuccetelli et al., 2020; Adams et al., 2020). This study therefore represents one of the most extensive of SARS-CoV2 evaluation studies where 30 LFIAs were evaluated over an eight month period.

For IgG, performance of the LFIAs were similar to sensitivities and specificities reported in available literature which ranged from 54% (95% CI: 44.4–62.3) to 100% (95% CI: 97–100) and 85% (95% CI: 76–91) to 100% (95% CI: 92.1–100), respectively (Bastos et al., 2020; Rashid et al., 2020; GeurtsvanKessel et al., 2020; Adams et al., 2020). In addition, the concordance between the LFIAs and RT-PCR was assessed by calculating the kappa co-efficient. The kappa co-efficient demonstrated fair to very good concordance (*What is Kappa coefficient (Cohen's Kappa*), n.d.) between the LFIA IgG and RT-PCR results. 4/30 (13%) of kits: (Zheihang Orient Gene COVID-19 IgG/IgM, Genrui Novel Coronavirus (2019-nCoV) IgG/IgM, Biosynex COVID-19 BSS IgG/IgM, Boson Biotech 2019-nCoV IgG/IgM) were recommended for SAHPRA approval based on kit sensitivity. Of these, only the Orientgene was recommended by SAHPRA (https://www.sahpra.org.za/) in August 2020 for use within the approved national testing algorithm while the remaining three received limited authorization for evaluation.

In terms of robustness, three kits demonstrated poor performance in terms of statistical measure and produced one, six and eight invalid result(s), respectively which are important factors for implementation consideration. Possible reasons for control band failures include insufficient testing volume, incorrect operating procedure or expired cassettes. All these possibilities could be excluded in this evaluation since a micropipette was used, the number of valid test results demonstrates operator competency and the cassettes were used within expiry dates by an experienced team.

For kits where a small volume (2 μl – 5 μl) of specimen is required, the package inserts recommends the use of a micropipette to ensure that the correct volume of specimen is added to the cassette. This presents a concern for implementation of this kit in the field where micropipettes are not readily available especially at point-of-care. An additional point to consider is that, specimen droppers provided with the majority of kits were often difficult to use which could have cost implications if specimen droppers need to be purchased separately.

Time to positivity for the different kits showed no relation with volume of specimen required or length of incubation and all kits had acceptable incubation periods for a rapid point-of-care test. Result interpretation was difficult for some kits where cassettes produced faint bands. False positive results are a concern for these kits since the package inserts state that “any shade of colour in the test region should be considered positive.” Some contained separate wells for the addition of specimen and diluent while others contained a single well for the addition of both.

Almost all kits detected IgM and IgG on the same cassette. The kit that offered separate cassettes for IgM and IgG testing had the following disadvantages compared to those where just one cassette was required: a larger volume of serum/plasma was required for testing and two tests required interpretation which translates to additional labour and time. The kit that detected IgG only has the obvious disadvantage that detection of IgM will be missed, if present in the patient specimen. Individual specimen ampules decreased risk of contamination however, the ampules were difficult to open, even with scissors and the diluent leaked out, causing spillage and a change of gloves between each test. A bulk specimen diluent contained in a single vial, however, has the disadvantage of cross-contamination and easily being misplaced.

Use of kits where testing can be performed on venous blood only requires a phlebotomist which will make implementation in a point-of-care setting difficult if fingerstick blood cannot be used. The fact that testing need to be performed within 30–60 min once the cassette is removed from its pouch needs to be taken into consideration for implementation at point-of-care sites where practice (for other kit types) often entails the unpacking of kits in advance to allow for easier use, when required. Of the 30 LFIAs evaluated, four were provided the status of SAHPRA approval (full or conditional) yet only one can be used as a standalone kit where required consumables will not need to be procured at an additional expense.

## Funding

EQUIP grant AID-OAA-A-15-00070 – Antiretroviral Therapy Simplification-Optimization of Programs and Services (ART-OPS) COVID supplement, and through iLEAD BMGF (i-LEAD) grant ID OPP1171455.
